# Light Mediated Generation of Silver Nanoparticles by Spinach Thylakoids/Chloroplasts

**DOI:** 10.1371/journal.pone.0167937

**Published:** 2016-12-09

**Authors:** Nisha Shabnam, P. Sharmila, Hyunook Kim, P. Pardha-Saradhi

**Affiliations:** 1 Department of Environmental Studies, University of Delhi, Delhi, India; 2 Department of Chemistry, Indian Institute of Technology Delhi, New Delhi, India; 3 Department of Energy and Environmental System Engineering, University of Seoul, Seoul, Korea; US Naval Research Laboratory, UNITED STATES

## Abstract

The unique potential of chloroplasts/thylakoids to harness light energy to transport electrons from H_2_O to various entities was exploited for reduction of Ag^+^ to generate nanoparticles (NPs). Spinach thylakoids/chloroplasts turned AgNO_3_ solutions brown in light, but not in dark. Besides showing Ag-NPs specific surface plasmon resonance band, these brown solutions showed presence of 5–30 nm crystalline NPs composed of Ag. Powder X-ray diffraction (PXRD) analysis revealed that Ag-NPs were biphasic composed of face-centered cubic Ag^0^ and cubic Ag_2_O. X-ray photoelectron spectroscopy (XPS) data further corroborated the presence of Ag_2_O in Ag-NPs. Limited formation of Ag-NPs in dark and increased generation of Ag^0^/Ag_2_O–NPs with increase in light intensity (photon flux density) by thylakoids/chloroplasts, established the role of light-harvesting photosynthetic machinery in generation of Ag^0^/Ag_2_O-NPs. Potential of thylakoids/chloroplasts to generate Ag-NPs from Ag^+^ on exposure to red and blue wavelength regions of visible light of electromagnetic spectrum, further confirmed the involvement of photosynthetic electron transport in reduction of Ag^+^ and generation of Ag-NPs. While light energy mediated photosynthetic electron transport donates energized electrons extracted from H_2_O to Ag^+^ to form Ag^0^-NPs, O_2_ released as a by-product during photolysis of H_2_O oxidizes Ag^0^ to form Ag_2_O-NPs. Our findings furnish a novel, simple, economic and green method that can be exploited for commercial production of Ag^0^/Ag_2_O-NPs.

## Introduction

Owing to unique properties, silver nanoparticles (Ag-NPs) find immense applications in engineering, medicine, agriculture and environment [1]. Due to excellent antimicrobial properties, there is a growing demand in use of Ag-NPs in hospitals, textiles, paints, emulsions, cosmetics etc. [1–3]. In spite of development of a number of green methods using biological systems or their components (which include extracts from various microbes/plants), none of the methods could replace chemical methods used for commercial production of NPs [4,5]. Although extracts of various plants/microbes are effective in generation of Ag-NPs, their use has been limited due to vast heterogeneity (in terms of size, shape and composition) of NPs generated. The vast heterogeneity results due to the presence of wide variety/range of biomolecules such as sugars, organic acids including amino acids, phenolics, soluble proteins etc., most of which have potential to generate metal nanoparticles [1–7].

Generation of Ag-NPs from Ag^+^ basically relies on reduction by reducing agents that have higher negative redox potential compared to Ag^+^ [6]. It is well established that light-harvesting photosynthetic machinery has immense potential to extract, energize and transport electrons from water to reduce various entities, which include NO_2_^-^, SO_4_^2-^ and O_2_, besides the key terminal acceptor, NADP^+^ [7,8]. This prompted us to evaluate if this potential of light-harvesting photosynthetic machinery to extract, energize and donate electrons can be exploited for reduction of Ag^+^. Here, we are reporting for the first time that light-harvesting photosynthetic machinery not only promotes reduction of Ag^+^ to Ag^0^ but also supports rapid oxidation of Ag^0^ to Ag_2_O by O_2_ released during photolysis of water to generate biphasic Ag-NPs.

## Materials and Methods

### Isolation of Thylakoids/Chloroplasts

Plants of spinach (*Spinacia oleracea* L.) were raised from seeds purchased from Agricultural Technology Information Centre (Pusa, New Delhi, India). Chloroplasts/thylakoids were isolated from spinach leaves as described earlier [7]. Leaves of *S*. *oleracea* were sliced into small pieces and incubated in isolation buffer (400 mM phosphate buffer with 5 mM NaCl, 1 mM MgCl_2_, 2 mM EDTA; pH 7.6) for 60 min in dark at 4°C. The leaves were then homogenized with chilled isolation buffer and filtered through 4 layers of Mira cloth. The filtered extract was centrifuged at 5000 xg at 4°C for 10 min and the resultant pellet containing thylakoids/chloroplast was suspended in distilled water instead of phosphate buffer as AgNO_3_ forms white precipitate with phosphate buffer. Thylakoids/Chloroplasts were washed 4 times with distilled water, through repeated suspension and centrifugation, to remove biomolecules like phenolics, sugars, free amino acids etc., which are known to promote generation of NPs [6,7,9]. The levels of chlorophyll, phenolics, sugars and amino acids were determined as described earlier [8,10].

### Evaluating Potential of Thylakoids/Chloroplasts to Generate Ag-NPs

4 mL reaction mixture consisting of 0, 0.1, 0.25 and 0.5 mM AgNO_3_ and thylakoids/chloroplasts equivalent to ~200 μg Chl was incubated under a photon flux density (PFD) of ~600 μmol m^-2^s^-1^ for different time intervals at 24±2°C. Another set of thylakoids/chloroplasts with AgNO_3_ was kept under similar conditions in dark.

For evaluating impact of light intensities on generation of Ag-NPs, thylakoids/chloroplasts were incubated with 0.5 mM AgNO_3_ under different PFDs (0, 60, 300, and 600 μmol m^-2^s^-1^), for different time intervals at 24±2°C. The absorbance of the samples was measured at 410 nm at regular time intervals.

For further confirming the involvement of light harnessing photosynthetic electron transport of isolated thylakoids/chloroplasts in reduction of Ag^+^ to generate Ag-NPs, the reaction mixture consisting of 0.5 mM AgNO_3_ and thylakoids equivalent to ~200 μg Chl were exposed to ~600 μmol m^-2^s^-1^ of red and blue light at 24±2°C. Blue and red light were supplied using 465 nm and 650 nm broad band filters [Swarovski Optik, USA (delivered by Advanced Research Scientific, India)], respectively.

### Characterization of NPs

NPs were characterized using UV-Vis spectrophotometer, transmission electron microscope (TEM) [coupled with energy dispersive X-ray (EDX) and selected area electron diffraction (SAED)] and powder X-ray diffraction (PXRD) as described earlier [7]. Chemical state of Ag-NPs was analyzed through X-ray photoelectron spectroscopy (XPS; Phi 5000 VersaProbe, Ulvac-Phi, Chigasaki, Japan).

### Establishing the Release of O_2_ by Thylakoids/Chloroplasts and its Role in generation of Ag_2_O-NPs

Potential of thylakoids to evolve O_2_ (i.e. PS II activity) was measured polarographically using Oxygraph enabled Clark Type liquid phase Oxygen electrode (Hansatech, UK). Reaction mixture (1 ml) containing thylakoids (equivalent to ~200 μg Chl) and 1μmole *p*-benzoquinone was illuminated with PFD of 600 μmol m^-2^s^-1^ and the rate of O_2_ evolution was measured. PS II activity was represented as μmol O_2_ evolved mg Chl^-1^ h^-1^.

To confirm that the O_2_ released by light-harvesting photosynthetic machinery of thylakoids/chloroplasts leads to oxidation of Ag^0^/Ag^0^-NPs to form Ag_2_O-NPs, investigations were carried under controlled anaerobic conditions. For achieving controlled anaerobic conditions (i) AgNO_3_ and other solutions used in this set of investigations were bubbled with saturated levels of N_2_ gas; (ii) vials containing the reaction mixture were capped and placed in a desiccator; (iii) air in this desiccator was displaced with N_2_ gas. Subsequently, the desiccator containing reaction the mixtures as well as respective control solutions was exposed to light of ~600 μmol m^-2^ s^-1^ PFD at 24±2°C.

All experiments were carried out independently at least six times and the data was subjected to Duncan’s multiple range test [11].

## Results and Discussion

**Fig 1:**
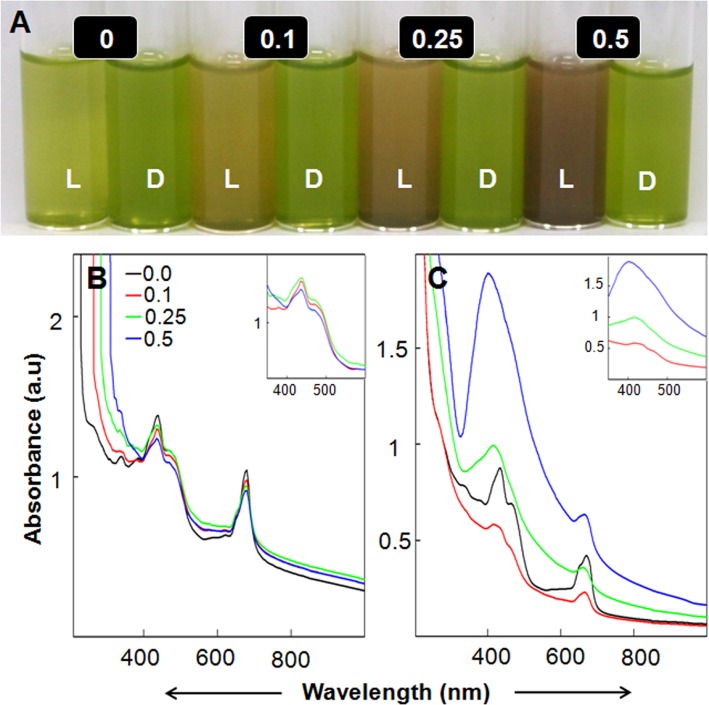
Potential of isolated thylakoids/chloroplasts to generate Ag-NPs. Variation in color of reaction mixtures containing different levels of AgNO_3_ (mM) incubated with isolated spinach thylakoids/chloroplasts in light (L) and dark (D) for 60 min (A). Absorption spectra of AgNO_3_ containing reaction mixtures incubated with spinach thylakoids/chloroplasts in dark (B) and light (C). Absorption band at ~410 nm in (C) corresponds to surface plasmon oscillations of Ag^0^/Ag_2_O-NPs. Insets show absorbance spectra of AgNO_3_ containing reaction mixtures incubated with thylakoids/chloroplasts in dark or in light on a magnified X-axis ranging from 350–630 nm. Absorption bands in the blue region at ~440 and 477 nm correspond to Chl *a* and Chl *b*, respectively. Absorption band at 470 nm correspond to carotenoids. Absorption bands at ~677 and 650 nm correspond to Chl *a* and Chl *b*, respectively.

Photosynthetic machinery has unique potential to harness light energy for extracting and energizing electrons from H_2_O for not only fixing CO_2_ but also for reduction of various entities associated with nitrogen and sulfur metabolism [6]. Isolated spinach thylakoids/chloroplasts turned AgNO_3_ solutions brown on exposure to light of ~600 μmol m^-2^ s^-1^ PFD within 60 min (Fig 1A). It is established that alteration in color of AgNO_3_ solution to brown is due to generation of Ag-NPs [2,3,12]. However, AgNO_3_ solutions incubated with thylakoids/chloroplasts in dark remained green (Fig 1). Absorption spectra of thylakoids/chloroplasts incubated in (i) light in absence of AgNO_3_ (control); and in (ii) dark in absence or presence of AgNO_3_, showed peaks specific for photosynthetic pigments [13,14] (Fig 1B and 1C). However, absorption spectra of AgNO_3_ solutions incubated with thylakoids/chloroplasts in light showed a prominent peak at ~410 nm (Fig 1C), which overlapped with peaks of photosynthetic pigments in this region. This specific peak around 410 nm, which arises due to surface plasmon oscillations of Ag-NPs [2,3,12], intensified with increase in AgNO_3_ concentration.

**Fig 2:**
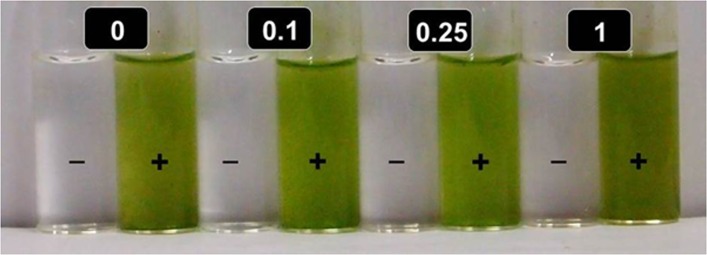
Thylakoids/chloroplasts must be photosynthetically active for generation of Ag-NPs in presence of light. Variation in color of reaction mixtures containing different levels of AgNO_3_ (mM) incubated without (-) and with (+) heat killed isolated spinach thylakoids/chloroplasts under a photon flux density of 600 μmol photons m^-2^ s^-1^ for 4 h.

AgNO_3_ solutions incubated without thylakoids/chloroplasts under identical conditions remained colorless both in light as well as dark (Fig 2). Since generation of Ag-NPs relies on reduction of Ag^+^, we believed that light energized photosynthetic machinery of spinach thylakoids/chloroplasts donates electrons to Ag^+^ to form Ag^0^ and Ag^0^-NPs. Heat killed thylakoids/chloroplasts failed to turn colorless AgNO_3_ solutions brown on incubation under identical conditions both in light as well as dark, further establishing involvement of photosynthetic machinery of live/active thylakoids/chloroplasts in generation of Ag-NPs (Fig 2).

**Fig 3:**
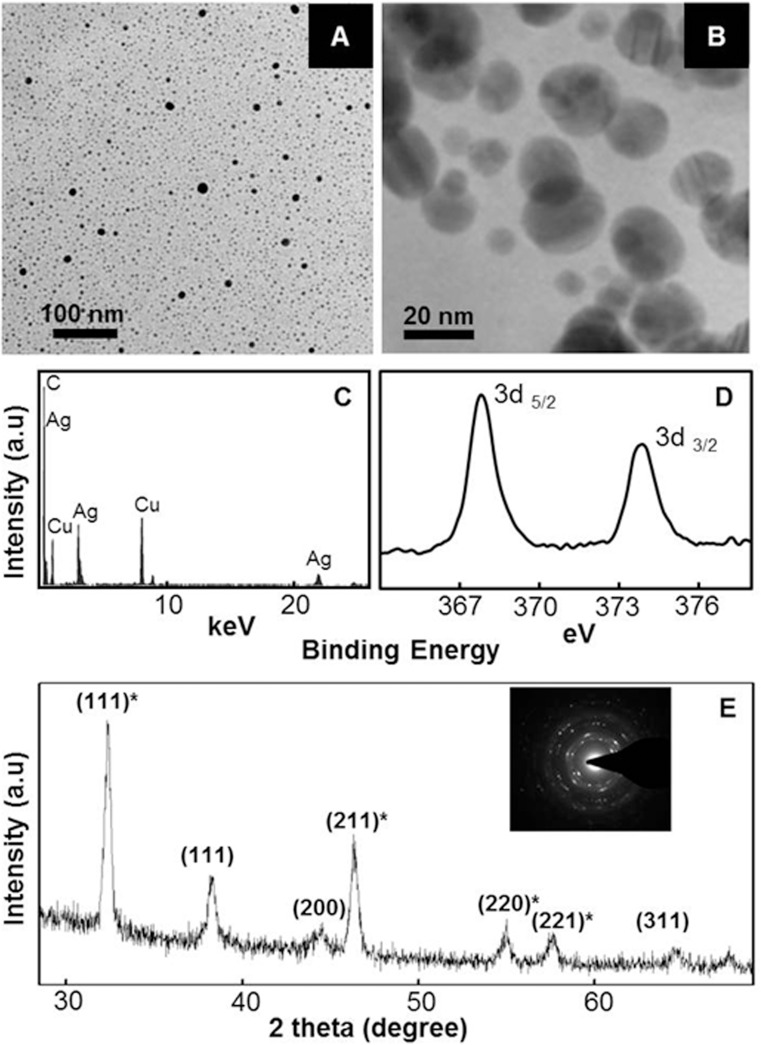
Characterization of Ag-NPs generated by thylakoids/chloroplasts in presence of light. TEM pictures (A-B); EDX spectrum (C); High resolution XPS spectrum (D); and PXRD pattern (E) of Ag^0^/Ag_2_O-NPs generated by spinach thylakoids/chloroplasts in presence of light. Inset in E represents selected area diffraction pattern of Ag^0^/Ag_2_O-NPs.

**Fig 4:**
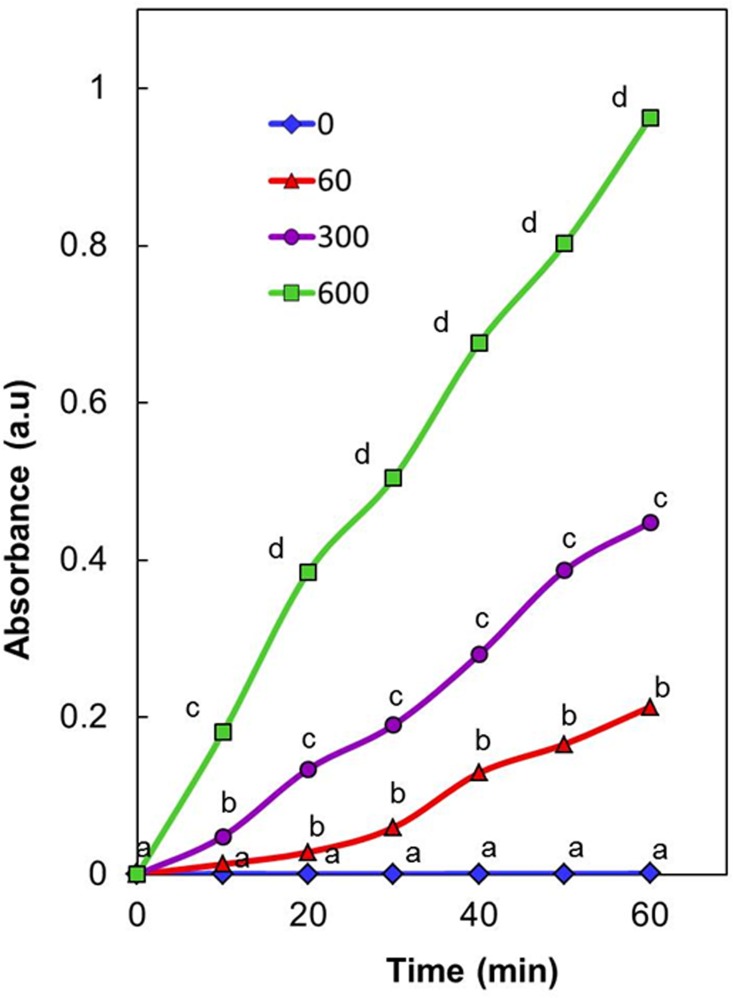
Impact of light intensity on potential of thylakoids/chloroplasts to generate Ag-NPs. Generation of Ag^0^/Ag_2_O-NPs by isolated spinach thylakoids/chloroplasts incubated in 0.5 mM AgNO_3_ exposed to light of varying photon flux density (μmol m^-2^s^-1^) for different time intervals. Values represent mean of data collected from six independent experiments. Values designated by different small letters are significantly different at *P*≤0.05 (Duncan’s multiple range test).

TEM investigations confirmed the presence of distinct NPs in brown solutions that resulted due to incubation of AgNO_3_ with thylakoids/chloroplasts in presence of light. Ag-NPs generated by thylakoids/chloroplasts were mostly spherical and in the size range of 10–30 nm (Fig 3A and 3B). EDX spectra showed the presence of distinct peaks which correspond to Ag (Fig 3C), conforming that NPs were composed of Ag. The PXRD pattern showed Bragg reflections (111), (200) and (311) revealing the face centered cubic structure and crystalline nature of Ag-NPs (Fig 3E). SAED pattern further corroborated that these NPs were crystalline in nature (inset in Fig 3E). Additional peaks seen in the PXRD spectra also corresponded to Bragg reflections (111)*, (211)*, (220)*, (221)* of cubic Ag_2_O (Fig 3E). Thus, PXRD studies clearly revealed that Ag-NPs were biphasic, composed of both Ag^0^ and Ag_2_O. It is well-known that Ag^0^ and Ag^0^-NPs are prone to oxidation [3,15,16] and thylakoids/chloroplasts generate O_2_ as a byproduct during extraction of electrons from water [7,8]. Potential of isolated spinach thylakoids/chloroplasts to evolve O_2_ is depicted in Fig 4. We also characterized Ag-NPs through XPS to further confirm the oxidation state of Ag. The high resolution XPS spectra showed two peaks arising due to emission of 3d_5/2_ and 3d_3/2_ photoelectrons at binding energies of ~367.7 and ~373.7 eV, respectively (Fig 3D). These peaks at the respective binding energies correspond to the values reported for Ag_2_O [17]. Thus, the XPS data clearly showed that Ag exists in +1 oxidation state (Ag^+^) i.e., in the form of Ag_2_O in these Ag-NPs, which is in corroboration with the PXRD data.

As photosynthetic electron transport is light intensity dependent, potential of thylakoids/chloroplasts to generate Ag^0^/Ag_2_O-NPs was evaluated under different PFDs, namely 60, 300, and 600 μmol photons m^-2^s^-1^ for different time intervals. The potential of thylakoids/chloroplasts to generate Ag^0^/Ag_2_O-NPs increased with increase in (i) PFD; and (ii) exposure duration (Fig 4). Increase in generation of Ag^0^/Ag_2_O-NPs with increase in PFD further established the involvement of photosynthetic electron transport in generation of Ag-NPs.

**Fig 5:**
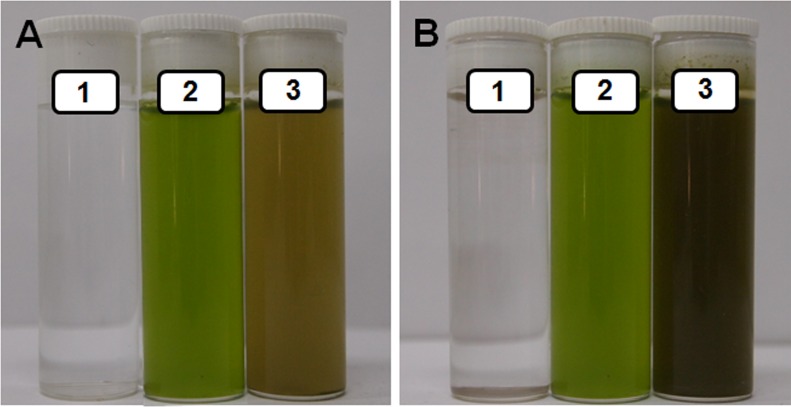
Potential of isolated thylakoids/chloroplasts to generate Ag-NPs in presence of red and blue light. Capacity of isolated spinach thylakoids/chloroplasts to generate Ag^0^/Ag_2_O nanoparticles from 0.5 mM AgNO_3_ on exposure to red (A) and blue (B) regions of visible light. 0.5 mM AgNO_3_ (1), thylakoids/chloroplasts incubated in absence (2) and presence (3) of 0.5 mM AgNO_3_ were exposed to ~600 μmol photons m^-2^ s^-1^ of red (650) and blue (465) wavelengths of visible light of electromagnetic spectrum.

It is well documented that red and blue wavelengths of the visible part of the electromagnetic spectrum are most effective for photosynthesis. Photosynthetic pigments absorb blue and red light more efficiently than the other spectral regions of photosynthetically active radiation [18]. Hence, overall photosynthetic electron transport linked to PS II quantum efficiency would be superior in red and blue regions of visible light. In order to further establish that photosynthetic electron transport of thylakoids/chloroplasts is involved in reduction of Ag^+^ and generation of Ag-NPs, we used broad band red (650 nm) and blue (465 nm) filters. As anticipated, we recorded alteration in color of green colored thylakoids/chloroplasts incubated with 0.5 mM AgNO_3_ to brown due to reduction of Ag^+^ and generation of Ag-NPs on exposure to red and blue wavelengths of light (Fig 5). These results further corroborated that light harnessing photosynthetic machinery of thylakoids is responsible for reduction of Ag^0^ and generation of Ag^0^/Ag_2_O-NPs.

**Fig 6:**
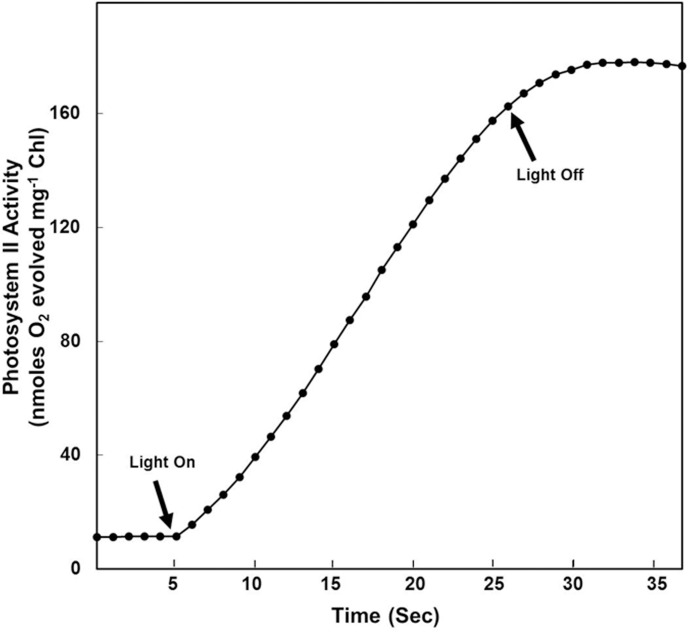
Potential of isolated thylakoids/chloroplasts to evolve O_2_. Potential of isolated spinach thylakoids/chloroplasts to evolve O_2_ rapidly on exposure to light of 600 μmol photons m^-2^s^-1^. Rate of photosystem (PS) II dependent O_2_ evolution was measured polarographically according to Shabnam et al. [8], using Oxygraph system enabled Clark type liquid phase O_2_ electrode (Hansatech Ltd., UK). Thylakoids/Chloroplasts equivalent to 20 μg of chlorophyll were used for each assay. It is evident that isolated thylakoids/chloroplasts evolve O_2_ only in presence of light.

**Fig 7:**
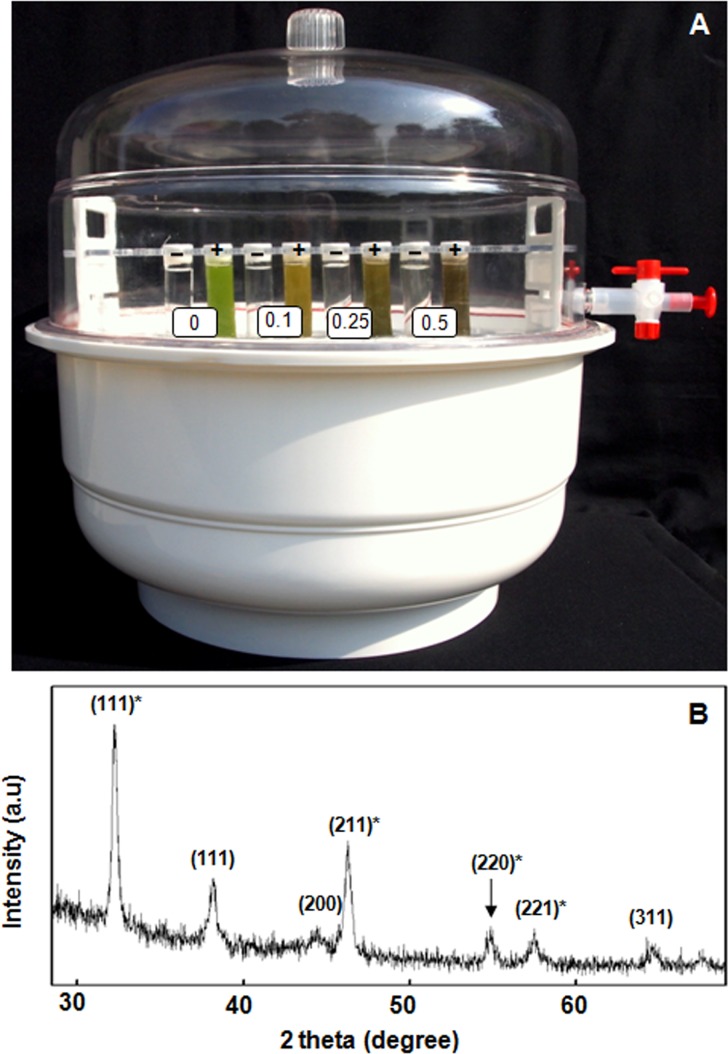
Thylakoids/chloroplasts are the primary source of O_2_ for generation of Ag_2_O-NPs in presence of light. Potential of isolated thylakoids/chloroplasts to generate Ag^0^/Ag_2_O-NPs under near controlled anaerobic conditions rapidly in presence of light of 600 μmol photons m^-2^s^-1^. Note variation in color of reaction mixtures containing different levels of AgNO_3_ (mM) incubated without (-) and with (+) isolated spinach thylakoids/chloroplasts (A). PXRD pattern (B) of Ag-NPs generated by spinach thylakoids/chloroplasts in presence of light. Bragg reflections (111)*, (211)*, (220)*, (221)* confirmed that large proportion of these NPs are composed of Ag_2_O.

It is well-known that light harvesting photosynthetic machinery of thylakoids/chloroplasts generate O_2_ as a byproduct during extraction of electrons from water [7,8]. We believe that Ag^0^ and Ag^0^-NPs, which are highly susceptible to oxidation [13,15,16], are readily oxidized by O_2_ released during photolysis of water, by light harvesting photosynthetic machinery of thylakoids. Potential of isolated spinach thylakoids/chloroplasts to evolve O_2_ in presence of light is depicted in Fig 6. In order to establish/confirm that photosynthetic machinery of thylakoids/ chloroplasts is the key source of O_2_ involved in oxidation of Ag^0^ and Ag^0^-NPs to form Ag_2_O-NPs, thylakoids incubated with AgNO_3_ solutions were maintained under near controlled anaerobic conditions in a desiccator (Fig 7A). Even under nearly anaerobic conditions, we recorded formation of NPs. PXRD analysis showed sharp Bragg reflections (111)*, (211)*, (220)* and (221)*, which revealed that the majority of these NPs were composed of Ag_2_O (Fig 7B). These results further established that it is the O_2_ released during photolysis of water by the light harvesting machinery of thylakoids/chloroplasts that plays key role in oxidation of Ag^0^ and Ag^0^-NPs to form Ag_2_O-NPs. It is well known that the light harvesting photosynthetic machinery is the key source of oxygen in the biosphere.

### Mechanism of Ag^0^/Ag_2_O-NPs Generation by Isolated Thylakoids/Chloroplasts

**Fig 8:**
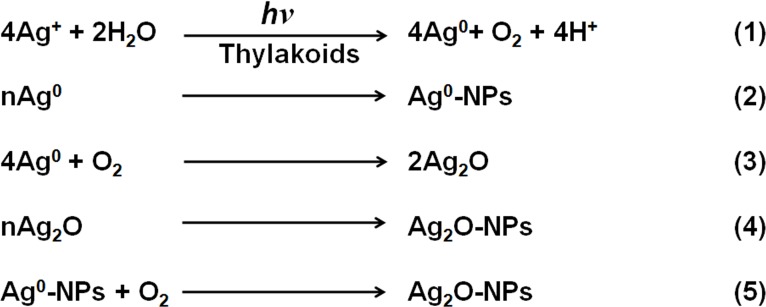
Steps involved in the synthesis of Ag^0^/Ag_2_O-NPs by isolated thylakoids/chloroplasts in presence of light. Basic reactions involved in generation of Ag^0^/Ag_2_O-NPs by isolated spinach thylakoids/chloroplasts of spinach using light energy.

Based on ultrastructural investigations that revealed prevalence of NPs in abundance in chloroplasts, earlier researchers believed the probable involvement of chloroplasts in generation of noble metal NPs [6,19,20]. Beattie and Haverkamp [6] believed that the reducing sugars produced in chloroplasts are responsible for the generation of noble metal-NPs. Although, we do believe that reducing sugars could have a role in generation of noble metal NPs in living cells, during present investigations, no detectable levels of sugars were seen in association with the isolated thylakoids/chloroplasts. Similarly, no traces of phenolics or amino acids were found in the isolated thylakoids/chloroplasts preparations used during present investigations. These observations convincingly demonstrated that the potential of isolated thylakoids/chloroplasts to generate Ag-NPs in presence of light is governed by the photosynthetic electron transport. Although, Dahoumane et al. [20] presumed participation of NAD(P)H-dependent reducing enzymes, like nitrate reductase, for generation of NPs in chloroplasts, we are strongly of the opinion that enzymes like nitrate reductase require specific substrates. Zhang et al. [21] reported generation of Au-NPs by isolated chloroplasts by stirring them in Au^3+^ solutions in water bath for 24–36 h and believed that proteins associated with chloroplasts were responsible for generation of Au-NPs. Contrary to earlier reports, our present investigations have clearly revealed the potential of thylakoids/chloroplasts to generate Ag-NPs in a short duration only in presence of light, clearly revealing the involvement of photosynthetic electron transport in generation of NPs through reactions depicted in Fig 8.

**Fig 9:**
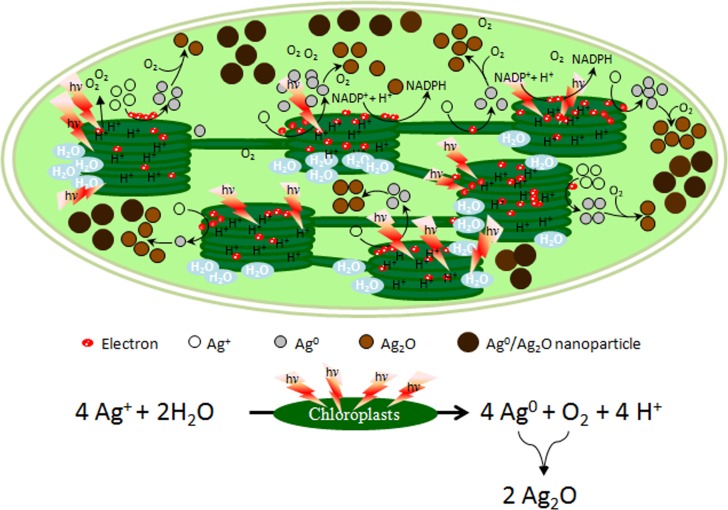
Mechanism of generation of Ag^0^/Ag_2_O-NPs by isolated thylakoids/chloroplasts in presence of light. A hypothetical model depicting mechanism of light mediated generation of Ag^0^/Ag_2_O-NPs by isolated spinach thylakoids/chloroplasts. Electrons extracted from water are energized and transported by light mediated photosynthetic electron transport system to Ag^+^ to reduce them and generate Ag^0^ and Ag^0^-NPs. O_2_, which is released as a byproduct during photolysis of water, readily oxidizes Ag^0^ as well as Ag^0^-NPs to generate Ag_2_O-NPs.

As photosynthetic electron transport machinery associated with thylakoids/chloroplasts, besides energizing and transporting the electrons (extracted from H_2_O) to the terminal acceptor NAD(P)^+^ by using light energy, can also transport electrons to other entities like O_2_, NO_2_^-^, SO_4_^2-^, etc. to bring about their reduction [7], it is clear that light driven photosynthetic electron transport initially promotes reaction 1, which releases O_2_ in addition to reduction of Ag^+^ to Ag^0^. It is widely believed that Ag^0^ generate Ag^0^-NPs (reaction 2) through steps involving nucleation and agglomeration [22] and may also get oxidized by the prevailing O_2_ to form Ag_2_O (reaction 3). Latter agglomerates to generate Ag_2_O-NPs (reactions 4 and 5). It is known that Ag^0^ and Ag^0^-NPs are highly prone to oxidation and form Ag_2_O-NPs under oxygenic conditions [3,14,15]. Therefore, we believe that O_2_ produced in the thylakoids/chloroplasts during photolysis of water readily oxidize Ag^0^ and Ag^0^-NPs to form Ag_2_O-NPs. A hypothetical model illustrating mechanisms of generation of Ag^0^/Ag_2_O-NPs from thylakoids/chloroplasts from Ag^+^ is depicted in Fig 9.

## Conclusions

Our findings clearly established that isolated thylakoids/chloroplasts can effectively generate Ag^0^/Ag_2_O-NPs using light energy. Light driven generation of Ag^0^/Ag_2_O-NPs by thylakoids/chloroplasts involves simple photochemical reactions involving (i) energization, transport and donation of electrons extracted from H_2_O through electron transport chain to Ag^+^ for its reduction to Ag^0^; (ii) spontaneous generation of Ag^0^-NPs through nucleation from Ag^0^; (iii) oxidation of Ag^0^ and Ag^0^-NPs to Ag_2_O-NPs by O_2_ released as a by-product during extraction of electrons from water. Light mediated generation of Ag^0^/Ag_2_O-NPs by thylakoids/chloroplasts is a novel, simple, economic and green method which can be exploited for large scale production of Ag-NPs, demand of which is growing rapidly.
